# Postexertional Supraventricular Tachycardia in Children with Catecholaminergic Polymorphic Ventricular Tachycardia

**DOI:** 10.1155/2012/329097

**Published:** 2012-07-04

**Authors:** Scott D. N. Else, James E. Potts, Shubhayan Sanatani

**Affiliations:** Division of Pediatric Cardiology, British Columbia Children's Hospital and The University of British Columbia, 1F9, 4480 Oak Street, Vancouver, BC, Canada V6H 3V4

## Abstract

Catecholaminergic polymorphic ventricular tachycardia (CPVT) is a severe arrhythmia associated with sudden death in the young. It is caused by defective calcium handling in ventricular myocytes. The association of supraventricular tachycardia (SVT) with CPVT is described in the literature, occurring in the lead-up to ventricular tachycardia during exercise testing. We describe three cases of SVT that were initiated in the recovery period of exercise testing in children with CPVT.

## 1. Introduction

Catecholaminergic polymorphic ventricular tachycardia (CPVT) is an uncommon condition causing sudden cardiac death in young, apparently healthy, individuals. CPVT is characterized by catecholamine-induced polymorphic or bidirectional ventricular tachycardia (VT) in patients with structurally normal hearts [1, 2]. The literature describes supraventricular tachycardia (SVT) in association with CPVT, usually occurring early in exercise testing, preceding VT [1]. We describe three cases of CPVT in which SVT was observed in the postexercise period.

**Case 1**

**Figure 1:**
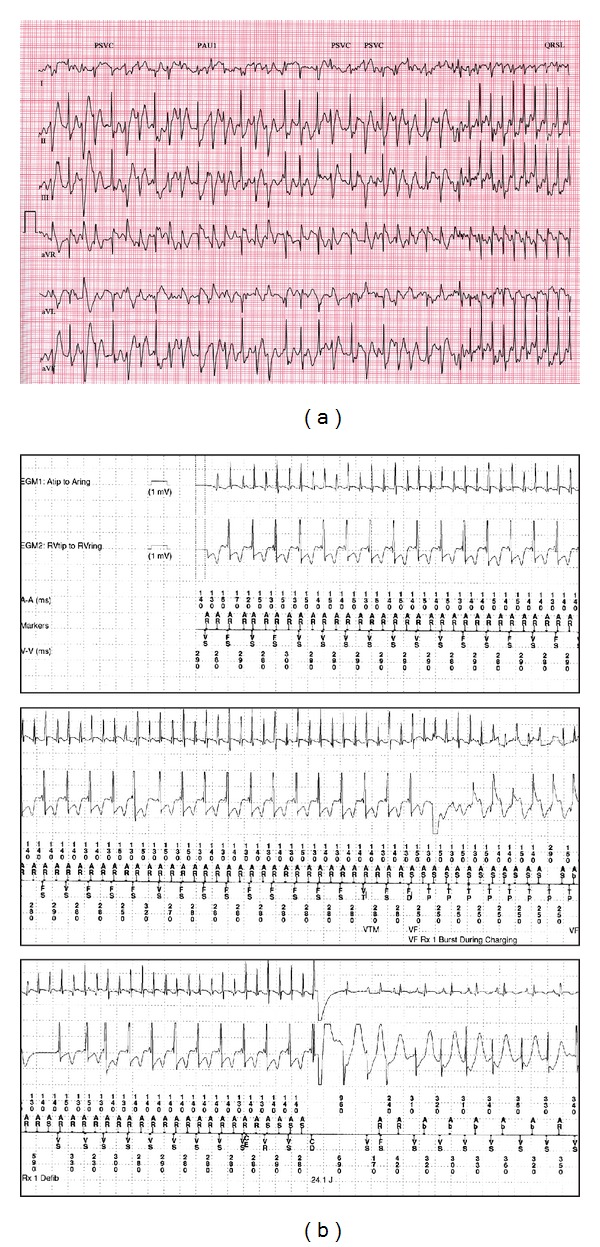
(a) Abrupt termination of bidirectional ventricular tachycardia with the onset of narrow complex tachycardia in the postexercise period (Case 1). (b) Electrograms from implantable defibrillator demonstrating 1 : 1 atrioventricular relationship during normal complex tachycardia (Case 1).

A 17-year-old boy presented with a 4-year history of syncope related to exertion or emotional stress. He had no other symptoms of cardiac disease, family history was noncontributory, and his cardiac examination was normal. His resting electrocardiogram (ECG) showed sinus bradycardia with a rate of 54 bpm and a corrected QT interval of 351 ms. His echocardiogram was normal. Twenty-four-hour Holter monitoring revealed <1% atrial ectopy and seven runs of SVT. An initial exercise test demonstrated a short run of atrial tachycardia as well as frequent ventricular ectopy. A repeat exercise test demonstrated frequent isolated premature ventricular contractions (PVCs) 10 minutes into exercise which progressed to bigeminy, then couplets and triplets. This bidirectional ventricular ectopy terminated abruptly in the immediate recovery period with the onset of SVT (Figure 1(a)). The patient was aware of these extrasystoles and the onset of SVT. He was maintained on beta blockers and, because he had had approximately 10 episodes of exertional syncope, he received a dual chamber implantable cardioverter defibrillator (ICD). Subsequently, his ICD discharged twice during exercise: once was an inappropriate shock for an episode of normal complex tachycardia and the other was an appropriate shock for a wide complex tachycardia. His normal complex tachycardia (Figure 1(b)) demonstrates a long ventriculoatrial interval, and, using his device, we demonstrated a lack of ventriculoatrial conduction at rest, suggesting that his normal complex tachycardia was conducting antegradely, though atypical retrograde conduction through the atrioventricular node was a possibility. He did undergo genetic testing for ryanodine mutations but no pathogenic mutation was found. He has been well controlled on a beta blocker with no further ICD discharges.

**Case 2**

**Figure 2:**
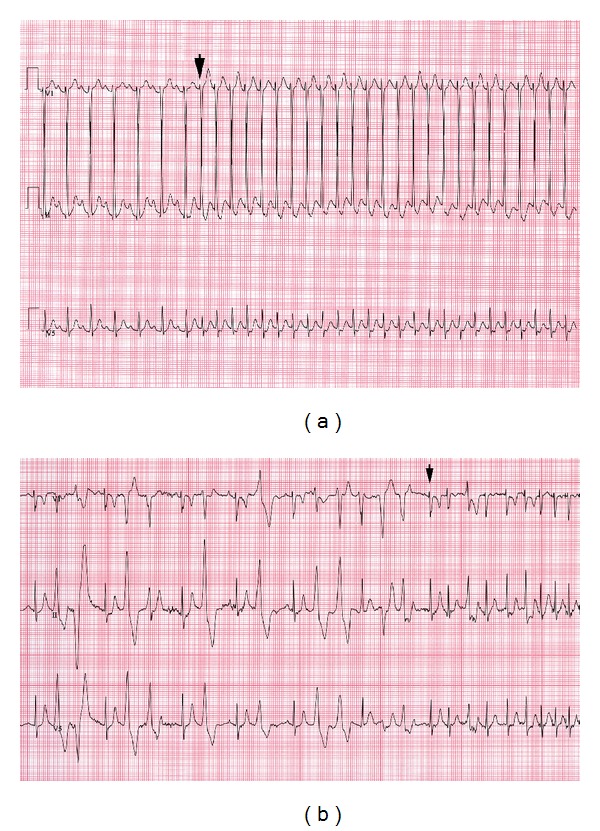
(a) Sudden onset of narrow complex tachycardia during the early recovery phase of exercise test (Case 2). (b) Bidirectional PVCs followed by couplets which give way to narrow complex tachycardia demonstrating warm-up of rate (arrow) early in recovery phase (Case 3).

A 13-year-old boy presented with a three-year history of exertional dizziness and palpitations; he denied syncope. He was otherwise healthy with no significant past medical or family history. His cardiovascular examination was normal. His resting ECG showed sinus bradycardia at 48 bpm and a corrected QT interval of 358 ms. Twenty-four-hour Holter monitoring showed ventricular ectopy at elevated heart rates including one 10 beat run of VT. His echocardiogram was normal and there was no evidence of arrhythmogenic right ventricular cardiomyopathy on his cardiac MRI. His exercise test demonstrated polymorphic PVCs which were tightly coupled to the preceding QRS. In the immediate recovery period he went into a narrow complex tachycardia with a variable rate of 180 to 230 bpm (Figure 2(a)); vagal maneuvers did not terminate the SVT. At this point he was started on Nadolol 40 mg daily and had complete resolution of his symptoms.

**Case 3**

A 6-year-old boy presented with a history of two episodes of exertional syncope. One episode happened during a karate class and the other while playing in the park. The patient's medical history was significant for dihydropyrimidine dehydrogenase deficiency, a genetic condition with no known cardiac manifestations. He had no known family history of syncope or sudden cardiac death. His cardiac examination was normal. Resting ECG showed sinus bradycardia with a corrected QT interval of 421 ms. Echocardiogram was normal. A Holter monitor was significant for frequent episodes of polymorphic VT that was often associated with exercise. Initial exercise testing showed bidirectional PVCs and nonsustained polymorphic VT. He was diagnosed with CPVT and started on Nadolol 40 mg daily. Subsequent exercise tests initially showed good suppression of his arrhythmia with only isolated PVCs. However, a recent exercise test showed deterioration of his condition with bidirectional PVCs, progressing to couplets, and then nonsustained VT. In the immediate recovery period the ventricular ectopy terminated abruptly with a run of SVT at approximately 200 bpm (Figure 2(b)). He was started on Flecainide 50 mg twice daily in addition to his Nadolol. Subsequent exercise testing showed suppression of his arrhythmia, with no atrial or ventricular ectopy on most recent testing. He has not had any further episodes of syncope.

## 2. Discussion

Patients with CPVT typically have normal cardiac structure and function and a resting ECG showing sinus bradycardia with a normal QT interval [3]. With exercise, there is a typical pattern of increasing ventricular ectopy with increasing heart rate [1, 3]. This progresses from isolated, monomorphic PVCs to salvoes of monomorphic and bidirectional VT [1, 3]. The typical morphology of the VT shows a beat-to-beat 180-degree alternation of the QRS axis reproducible with treadmill testing [3]. Disordered calcium handling is thought to be responsible for this arrhythmia with adrenergic stimulation of the cardiac myocytes causing augmentation of calcium influx and diastolic calcium spikes [4]. 

Given this mechanism, it follows that atrial arrhythmias, in addition to ventricular arrhythmias, would be seen in patients with CPVT. Leenhardt et al. reported 21 patients with CPVT and described 4 with “bursts of atrial tachyarrhythmia” (atrial fibrillation and junctional tachycardia) [1]. Typically, the onset of atrial arrhythmias is observed at lower heart rates than ventricular arrhythmias in patients with CPVT [1, 5]. It has been postulated that atrial arrhythmias may be the precursor to VT [4]. The onset of SVT occurred in the recovery period of the exercise test in our patients. It is possible that, in at least one of our patients (Case 3), the ventricular ectopy may have contributed to the development of SVT. As all three patients have the phenotype of CPVT, it is most likely that disordered calcium handling and increased sensitivity to catecholamines underlies this process. Genetic testing has not been carried out in all of our patients.

In summary, we described three cases of children with CPVT in whom SVT was observed in the recovery phase of exercise testing. To our knowledge, this association has not been reported before and represents a new dimension to this complex arrhythmia substrate.
